# Bile Acid Metabolism as a Unifying Readout for Diet, Microbiome Function, and Gastrointestinal Disease

**DOI:** 10.1096/fj.202602616RR

**Published:** 2026-09-03

**Authors:** Jason Dichter

**Affiliations:** ^1^ University of Pittsburgh Pittsburgh Pennsylvania USA

**Keywords:** bile acids and salts, colorectal cancer, dietary fiber, fermented foods, FXR, gastrointestinal microbiome, irritable bowel syndrome, microbially conjugated bile acids, polyphenols, primary biliary cholangitis

## Abstract

Bile acid biology has advanced through significant conceptual shifts. Once understood primarily as biological detergents, bile acids are now recognized as signaling molecules and, more recently, as a chemically diverse set of host‐ and microbe‐derived metabolites. The 2020 discovery of microbially conjugated bile acids (MCBAs) and the expansion of the recognized catalog from approximately 20 species to more than 200 mark a new phase in this trajectory. Clinical translation has not kept up. Direct farnesoid X receptor (FXR) agonism failed twice in trials for non‐alcoholic steatohepatitis, later received a serious liver injury safety communication from the U.S. Food and Drug Administration, and was voluntarily withdrawn from the United States market. The standard of care for bile acid diarrhea and post‐cholecystectomy diarrhea still relies largely on chemical binding with drugs introduced in the 1960s and 1970s. This Perspective argues that the gap between bile acid biology and bile acid medicine persists in part because the field has not been organized around the bile acid pool as a shared measurable output. Researchers studying dietary modulators of the gut microbiome have worked in separate communities around fiber, fermented foods, polyphenols, protein, and dietary fat. Each of these inputs can shape bile acid metabolism, yet many intervention studies do not measure it. The most immediately implementable bile acid–targeted strategy is specified dietary intervention designed with measurable bile acid outcomes and evaluated with the precision of pharmacological therapy. Four recommendations follow: intervention studies should routinely measure bile acid outcomes; clinicians should test bile acid metabolism in conditions involving dysregulation; regulators should develop a framework for multicomponent dietary therapies; and researchers should build infrastructure for population‐scale longitudinal monitoring.

## Graphical abstract

Dietary components shape the gut microbiome, which modifies bile acids through transformations. The resulting bile acid pool is sensed by host receptors to regulate immunity, metabolism, and intestinal function. Targeted bile acid measurements provide a unifying readout to study diet‐microbiome interactions and to develop next‐generation dietary therapeutics for gastrointestinal and liver diseases.

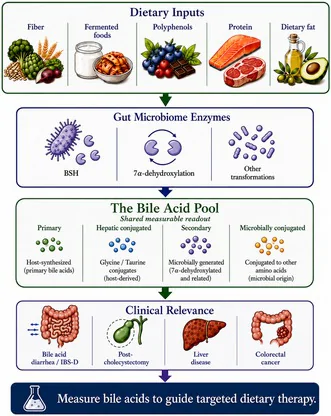

**Abbreviations**
ASBT: apical sodium‐dependent bile acid transporter
BAD: bile acid diarrhea
BSH: bile salt hydrolase
FGF19: fibroblast growth factor 19
FMT: fecal microbiota transplantation
FXR: farnesoid X receptor
IBS‐D: diarrhea‐predominant irritable bowel syndrome
LC–MS: liquid chromatography‐mass spectrometry
MCBA: microbially conjugated bile acid
OCA: obeticholic acid
PBC: primary biliary cholangitis
PPAR: peroxisome proliferator‐activated receptor
SCFA: short‐chain fatty acid
TGR5: Takeda G‐protein‐coupled receptor 5
UDCA: ursodeoxycholic acid

## 1 The Translational Gap in Bile Acid Biology

Bile acid biology has undergone several substantial transformations. Bile acids were first understood primarily as biological detergents required for lipid absorption. The discovery that bile acids also act as signaling molecules through receptors such as farnesoid X receptor (FXR) and Takeda G protein‐coupled receptor 5 (TGR5) marked a major earlier shift in the field [1, 2, 3, 4]. The last 6 years have opened a newer phase of that history. Bile acids are now being recognized not only as host‐derived digestive and signaling molecules but as a chemically diverse set of metabolites continuously shaped by host and microbial enzymes. In 2019, Hang and colleagues identified isoallolithocholic acid and 3‐oxolithocholic acid as regulators of regulatory T‐cell and T‐helper 17‐cell differentiation [5]. This finding connected microbial bile acid metabolism directly to immune balance. In 2020, Quinn, Dorrestein, and colleagues discovered microbially conjugated bile acids (MCBAs) [6], a class of molecules that challenged the long‐standing assumption that bile acid conjugation occurred only in the liver through bile acid–coenzyme A:amino acid N‐acyltransferase (BAAT). MCBAs showed that gut microbes can carry out related chemistry using a wider range of amino acids than glycine and taurine. In the years since, the catalog of microbially modified bile acid species has expanded from roughly 20 known species to more than 200 [7], and MCBAs in human stool have been estimated to exceed classical glycine and taurine conjugates in total concentration by approximately threefold [7, 8]. The bile acid pool, once treated as a relatively closed set of fat‐digesting molecules with predictable effects, is now better understood as a dynamic chemical landscape shaped by both host physiology and microbial metabolism.

Clinical translation has not kept pace with this mechanistic resolution. The leading modern attempt to drug bile acid signaling directly was obeticholic acid (OCA), a synthetic FXR agonist. OCA was rejected by the Food and Drug Administration (FDA) for non‐alcoholic steatohepatitis, now termed metabolic dysfunction‐associated steatohepatitis (MASH), in 2020 and again in 2023. The drug met histologic improvement endpoints in the REGENERATE trial, but the FDA judged that histologic change alone did not establish clinical benefit at a level sufficient for approval [9, 10]. In December 2024, the U.S. Food and Drug Administration issued a safety communication warning of serious liver injury, including cases requiring transplantation, in patients without cirrhosis taking OCA for primary biliary cholangitis (PBC) [11]. In November 2025, OCA was voluntarily withdrawn from the United States market for its remaining indication in PBC, an autoimmune liver disease in which the bile ducts are gradually destroyed [12]. This trajectory does not show that FXR is an invalid therapeutic target. It does show that direct receptor pharmacology has not yet converted modern bile acid biology into a durable clinical platform. Meanwhile, the standard treatment for bile acid diarrhea (BAD) remains chemical sequestration with cholestyramine, colestipol, and colesevelam, drugs introduced in the 1960s and 1970s. BAD affects approximately 25%–35% of patients diagnosed with diarrhea‐predominant irritable bowel syndrome (IBS‐D) [13, 14], and is identified in roughly 60% of post‐cholecystectomy patients when those patients are investigated for diarrhea [15]. The role of secondary bile acids in colorectal cancer biology has also been studied for decades [16], yet this mechanistic knowledge has not produced bile acid‐guided dietary or microbial prevention strategies in standard clinical guidelines.

Reasonable observers will note that the field has not ignored these problems. Drugs that activate the peroxisome proliferator‐activated receptors (PPARs), including elafibranor and seladelpar, are emerging as second‐line therapies for PBC [17, 18]. Clinical trials of probiotics in cholestatic liver disease and inflammatory bowel disease have produced encouraging mechanistic signals [19], and the success of fecal microbiota transplantation (FMT) along with defined microbial consortia in recurrent Clostridioides difficile infection [20, 21] demonstrates that microbiome‐based therapeutics can reach the clinic. These are real advances, so the argument here is not that the field has been inactive. The argument is that its activity has often been organized around receptors, organisms, diets, or diseases rather than around the bile acid pool as a shared measurable output.

**FIGURE 1:**
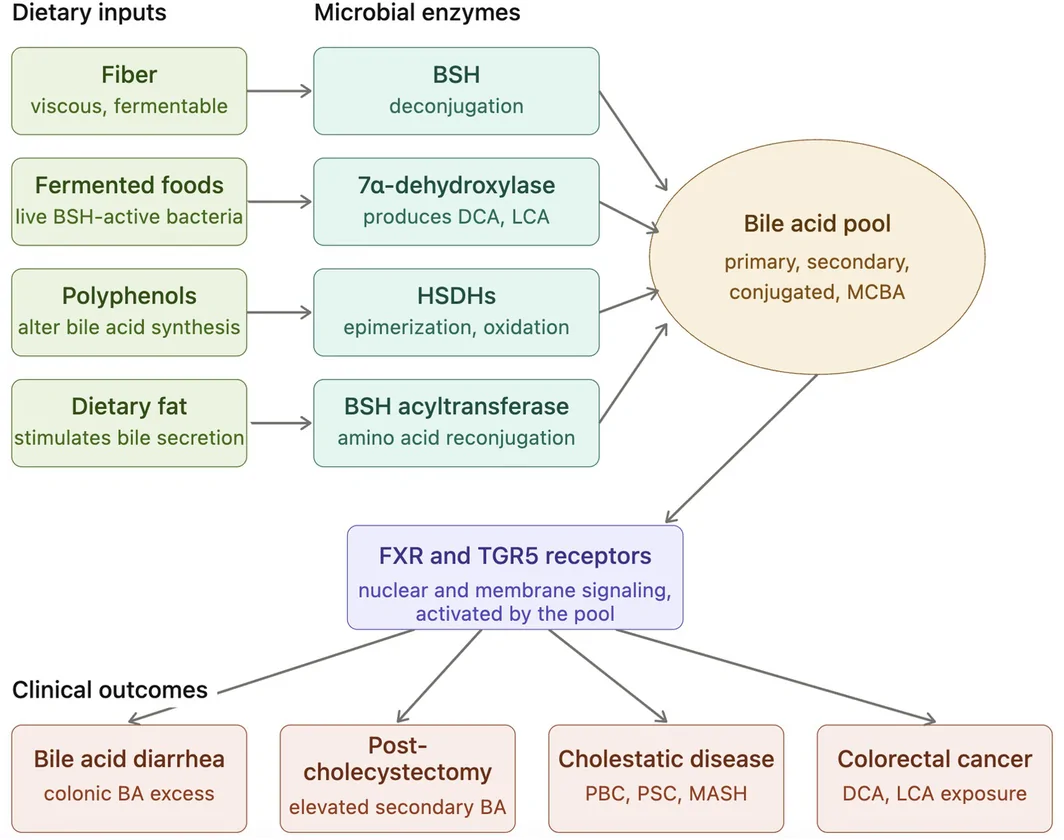
The bile acid pool as a convergence point linking dietary inputs to clinical outcomes. Four classes of dietary input (green) act on distinct microbial enzymes (teal) that reshape the composition of the gut bile acid pool (amber). BSH cleaves glycine or taurine from conjugated bile acids; 7α‐dehydroxylase produces the secondary bile acids deoxycholic acid (DCA) and lithocholic acid; hydroxysteroid dehydrogenases (HSDHs) drive epimerization and oxidation reactions; BSH acyltransferase reconjugates bile acids with novel amino acids to form microbially conjugated bile acids (MCBAs). The pool acts on the host through the nuclear receptor FXR and the membrane G‐protein receptor TGR5 (purple), producing four representative clinical outcomes (coral): Bile acid diarrhea (BAD), post‐cholecystectomy syndromes, cholestatic liver diseases including primary biliary cholangitis (PBC), primary sclerosing cholangitis (PSC), and metabolic dysfunction‐associated steatohepatitis (MASH), and colorectal cancer. Five nutritional literatures share this common downstream variable. This is a conceptual schematic, and arrows indicate principal demonstrated relationships and are not exhaustive.

The central opportunity is bile acids themselves. Researchers studying dietary modulators of the gut microbiome have worked largely in separate communities around fiber, fermented foods, polyphenols, protein source, and dietary fat composition. Each of these dietary inputs can influence bile acid metabolism (Figure 1), but too few studies measure bile acid outcomes directly. This Perspective advances two claims. First, bile acids deserve to be treated as a central output in research connecting diet, the gut microbiome, and disease. The model for this transition is how short‐chain fatty acids (SCFAs), produced when gut bacteria ferment dietary fiber, became a standard set of molecules used to interpret fiber effects over the last 2 decades [22]. Second, the most immediately implementable bile acid–targeted therapeutic strategy may be dietary rather than pharmacological. This strategy involves specified dietary interventions designed with measurable bile acid outcomes and evaluated with the precision expected of pharmacological therapy rather than the vagueness of lifestyle advice. The biology supports this framing; the clinical need is real, and the remainder of this Perspective makes the case for measuring bile acids as the common output through which disconnected dietary literatures can become clinically testable.

## 2 What the Bile Acid Pool Actually Tells You

Bile acids are produced in hepatocytes from cholesterol via two pathways: the classical (initiated by cholesterol 7α‐hydroxylase) and the alternative (initiated by sterol 27‐hydroxylase). Both converge on the primary bile acids cholic acid and chenodeoxycholic acid, which are then conjugated by BAAT to glycine or taurine before secretion into bile [23]. Roughly 95% of conjugated bile acids entering the small intestine are recovered at the terminal ileum through the apical sodium‐dependent bile acid transporter (ASBT) and returned to the liver via portal circulation [23]. The remaining 5% escapes reabsorption and reaches the colon, the most chemically diverse part of the gastrointestinal tract because of its dense microbial community [23]. FXR activation in ileal enterocytes induces fibroblast growth factor 19 (FGF19), which travels back to the liver and suppresses CYP7A1 transcription, slowing further bile acid synthesis [4]. Considered on its own, the host side of this circuit is closed and stable; variability, complexity, and most of the clinical interest enter through the microbial side.

Bile salt hydrolases (BSHs) are enzymes distributed broadly across the gut microbiota and particularly enriched in *Lactobacillus* and *Bifidobacterium*, along with *Bacteroides* and *Clostridium* species [24, 25]. These enzymes cleave the amide bond that connects the bile acid backbone to its attached amino acid, releasing what are called unconjugated primary bile acids. This step, known as deconjugation, is the gateway for nearly all subsequent microbial chemistry on bile acids. Once deconjugated, primary bile acids become substrates for further transformation. The most consequential of these transformations is 7α‐dehydroxylation, a multi‐step reaction carried out by *Clostridium scindens* and related species, which produces the secondary bile acids deoxycholic acid (DCA) and lithocholic acid. Other enzymes called hydroxysteroid dehydrogenases, distributed across many additional bacterial species, modify the bile acid structure further by changing the orientation or oxidation state of specific chemical groups, generating what are known as iso, allo, and oxo derivatives [23, 25]. Together, these reactions can interconvert dozens of distinct bile acid species and dramatically shift how strongly the pool binds to its receptors, how soluble it is in water, and how biologically active it becomes.

Until 2020 this account of microbial bile acid chemistry was considered essentially complete. It is now understood to have been a substantial underestimate. The 2020 identification of bile acids that had been reconjugated by gut microbes to amino acids other than glycine or taurine [6] introduced a category of molecules with no known host equivalent. These new molecules, MCBAs, were initially identified as conjugates of phenylalanine, tyrosine, and leucine, but further work has expanded the catalog significantly. MCBAs have now been identified as conjugates with valine and 5‐aminovaleric acid, with the neurotransmitter *γ*‐aminobutyric acid (GABA), and with 2‐aminobutyric acid as well as additional standard amino acids [7, 8]. A separate class of MCBAs is formed by attachment of short‐chain fatty acids at a different position on the bile acid molecule called the 3‐hydroxyl group [8]. Most recently, in 2024, a bile acid conjugated with succinate (a molecule produced during cellular energy metabolism) was identified as a protective metabolite for the liver. This molecule, 3‐succinylated cholic acid, acts through a separate bile acid receptor, TGR5 [26]. The enzyme responsible for much of this chemistry has been traced back to BSH itself, which is now understood to catalyze both deconjugation and reconjugation depending on which substrates are available [27]. This dual function of a single enzyme escaped notice for decades because researchers had not thought to look for the reverse reaction.

Three properties make the bile acid pool an unusually informative variable for research connecting diet, the gut microbiome, and disease. First, it sits downstream of both host and microbial inputs, and it integrates them quantitatively in a single chemical readout. A dietary intervention that changes microbial community composition will leave a measurable fingerprint in the bile acid pool whether or not the microbial change is directly visible in standard sequencing data. Second, the pool is distributed along the digestive tract in ways that map onto specific diseases. Bile acid concentrations in the small intestine matter for FXR signaling and feedback control of bile acid synthesis. Bile acid concentrations in the colon matter for the role of secondary bile acids in colorectal cancer and BAD. Bile acid concentrations in the bloodstream matter for cholestatic liver diseases such as PBC, along with metabolic liver disease. Third, the chemistry is measurable at scale. Targeted liquid chromatography–mass spectrometry (LC–MS) panels can now quantify 30 to 50 bile acid species reliably, and untargeted metabolomics is steadily extending detection to the broader array of MCBAs [7, 8].

One feature of bile acid signaling deserves explicit emphasis because it shapes therapeutic strategy directly. Signaling at FXR and TGR5 is what biologists call phasic, meaning the effects are not the same across all concentrations. Instead, the effects depend on how much bile acid is present. At normal physiologic concentrations, bile acids activate these receptors with effects that support cell growth and gut barrier integrity, along with beneficially regulating the immune system. At higher concentrations, the same molecules become damaging to cell membranes and toxic to mitochondria and trigger apoptosis [28]. Higher luminal or systemic bile acid exposures can occur after high‐fat meals, in cholestatic liver disease, and after gallbladder removal. Therapeutic FXR activation must therefore be considered alongside the concentration‐dependent toxicity of bile acids and bile acid‐like ligands. This is not an academic detail; the OCA experience illustrates how receptor‐directed bile acid pharmacology can face safety constraints in the same disease populations where therapeutic activation is being pursued [11, 29]. It is also the reason microbial modulation of the bile acid pool, which adjusts the composition of the pool rather than driving any single receptor to full activation, is an inherently more plausible long‐term therapeutic strategy. Figure 1 summarizes this convergence: dietary inputs act through microbial enzymes to shape the bile acid pool, which engages FXR and TGR5 to produce a range of clinical outcomes.

## 3 How Diet Shapes the Bile Acid Pool

The main claim of this Perspective is that major dietary modulators of the gut microbiome may share an undermeasured downstream variable: the bile acid pool. Nutrition science has not yet integrated this insight across the major dietary literatures. Table 1 summarizes these dietary inputs, their mechanisms, and their effects on the bile acid pool. These four were selected because each has a substantial published literature on gut microbiome modulation and at least preliminary evidence of bile acid pool effects, whereas other dietary inputs likely also matter but have less direct mechanistic evidence to date.

**TABLE 1:** Dietary inputs and their bile acid signatures across five nutritional research literatures. Each category of dietary input shapes the gut bile acid pool through distinct mechanisms, with downstream effects on cardiovascular, metabolic, and gastrointestinal disease. Five disconnected research communities (fiber science, fermented food microbiology, polyphenol pharmacology, dietary fat epidemiology, and protein source comparisons) share this common output variable. Current research designs in each field rarely measure bile acid changes as a unifying mechanism.

Dietary input	Primary mechanism	Examples	Bile acid pool shift	Clinical relevance
Fiber (viscous)	Sequesters bile acids in small intestine, increases fecal loss, upregulates hepatic CYP7A1	*β*‐glucan, psyllium, pectin, guar gum	↑ fecal BA loss ↑ hepatic synthesis ↓ serum LDL	Cardiovascular risk reduction; cholestasis adjunct
Fiber (fermentable)	Reshapes microbial community, alters BSH and 7α‐dehydroxylating populations	Inulin, resistant starch, raffinose	Altered conjugation balance ↑ hepatic taurine conjugates	Metabolic and inflammatory bowel conditions
Fermented foods	Delivers live BSH‐active organisms, expands microbial deconjugation capacity	Yogurt, kefir, sourdough, kimchi, miso	↓ conjugated BA ↑ unconjugated and secondary BA	Cholesterol metabolism; bile acid diarrhea; cholestasis adjunct
Polyphenols	Modulates CYP7A1 expression, shifts microbial composition toward BSH‐active species	Tea catechins, grape flavonoids, berry anthocyanins	↑ BA synthesis ↑ fecal BA excretion	Lipid lowering; cardiometabolic prevention
Dietary fat (Western pattern)	Drives sustained bile secretion, selects for bile‐tolerant bacteria and 7*α*‐dehydroxylators	Saturated fat, high total fat intake	↑ DCA, LCA ↑ colonic secondary BA exposure	Colorectal cancer risk; metabolic liver disease
Protein source	Animal protein favors taurine conjugation; plant favors glycine; alters H_2_S production	Red meat vs. legumes, animal vs. plant protein	Taurine vs. glycine shift ↑ H_2_S with animal protein	Colorectal cancer; inflammatory disease

*Note:* ↑, increased; ↓, decreased.

Abbreviations: BA, bile acid; BSH, bile salt hydrolase; CYP7A1, cholesterol 7α‐hydroxylase; DCA, deoxycholic acid; H_2_S, hydrogen sulfide; LCA, lithocholic acid; LDL, low‐density lipoprotein; MCBA, microbially conjugated bile acid.

### 3.1 Dietary Fiber

The bile acid–lowering effect of soluble fiber is one of the oldest mechanistically validated diet–disease relationships in clinical nutrition [30]. Viscous and adsorptive fibers bind bile acids in the small intestinal lumen, prevent their reabsorption at the terminal ileum, and force their excretion in stool. The liver compensates for this loss by synthesizing new bile acids from cholesterol, which depletes the liver's cholesterol pool and increases the activity of low‐density lipoprotein receptors that pull more cholesterol out of the blood. This bile acid–driven mechanism is responsible for the cholesterol‐lowering effect of psyllium, oat *β*‐glucan, and pectin.

This framework captures only half of what dietary fiber does to the bile acid pool. The other half is microbial. Studies in mice fed standardized diets containing nine fiber types, including cellulose, chitin, resistant starch, pectin, inulin, *β*‐glucan, psyllium, dextrin, and raffinose have shown that fiber type produces distinct microbial community signatures and, thus, distinct bile acid profiles [31]. Inulin and *β*‐glucan in particular produce a striking pattern: taurine‐conjugated bile acids are elevated in the liver but reduced in the intestine, a distribution most easily explained by enhanced microbial BSH activity in the gut [31]. These fermentable fibers may enrich the BSH‐bearing community and shift the bile acid pool toward greater deconjugation and downstream secondary bile acid formation. This mechanism is distinct from the sequestration pathway and produces distinct clinical effects [31].

The bile acid sequestration mechanism of viscous, less fermentable fibers such as psyllium and certain pectin formulations drives cardiovascular benefit, and this mechanism is best preserved when the fiber survives transport to the terminal ileum without being fermented away. The BSH‐stimulation mechanism proposed for fermentable fibers such as inulin and *β*‐glucan, along with certain resistant starches, may produce a different set of effects through shifts in the microbial bile acid pool. These effects are relevant to bile acid–driven inflammation, colorectal cancer risk, and how the body handles bile acids after gallbladder removal. The implication is that current dietary guidelines, which bundle fiber into a single recommendation, miss the granularity that bile acid measurement would make visible.

### 3.2 Fermented Foods

Fermented foods are the most direct dietary input to microbial bile acid chemistry, and they are also the input where the gap between mechanistic possibility and human clinical evidence is widest. BSH activity is one of the defining biochemical properties of food‐associated *Lactobacillus* and *Bifidobacterium* strains, and it has historically served as a selection criterion for probiotic candidates [24, 32]. Food‐fermented *Lactobacillus plantarum* strains withstand the stress of bile exposure and grow in bile‐rich environments, while also selectively altering bile acid signatures in directions that favor host receptor activation [33]. The dual function of BSH as both a deconjugating enzyme and a re‐conjugating enzyme [27] places fermented food bacteria not at the periphery of bile acid chemistry but at its center: the same enzyme that removes amino acids from bile acids also generates much of the MCBA catalog described in Section 2.

Sourdough bread provides an instructive example, but it should be framed as a candidate model rather than established evidence for bile acid modulation. Baking eliminates viable fermenting organisms from the final product, so sourdough should not be treated as a fermented food that works primarily by delivering live bacteria. Its relevance instead lies in the chemical products of fermentation. During fermentation, lactic acid bacteria and yeasts generate organic acids, bioactive peptides, exopolysaccharides, and modified plant phenolics, some of which may persist after baking and plausibly influence host or microbial metabolism after ingestion [34]. Whether these effects include reproducible changes in human bile acid profiles remains an open question. For that reason, sourdough is useful as a model for future bile acid–focused dietary intervention studies.

Greek yogurt works through a related but distinct mechanism. Unlike sourdough, yogurt delivers live bacteria, including organisms that may express bile salt hydrolase activity. Its downstream effects on bile acid metabolism should therefore depend on strain composition, survival through gastrointestinal transit, and the bile acid‐transforming capacity of the organisms delivered. Yogurt is also a useful research vehicle because its bacterial content is concentrated and its composition is more reproducible across batches than many other fermented foods [35]. The contrast between sourdough and yogurt illustrates the broader point: fermented foods should not be treated as one mechanistic category. Some deliver live BSH‐active organisms; others deliver fermentation‐derived chemical products. Both may be relevant to bile acid biology, but they require different study designs and different endpoints.

Where the case weakens is in human evidence. The most rigorous Human Trial I am aware of compared three single‐strain probiotics, namely *Bacillus subtilis* , *L. plantarum* , and *Bifidobacterium animalis* subspecies *lactis* against placebo in healthy adults over 18 weeks [36]. Each strain produced a distinct pattern of changes in plasma bile acids, but the size of each effect was small, and the direction of change varied unpredictably across strains. This trial demonstrates that probiotic‐associated changes in plasma bile acid profiles can be measured in humans, while also showing how limited the human intervention literature remains. Few probiotic trials include bile acid measurements, and few bile acid studies use fermented food interventions. The result is a literature in which the underlying biology is well‐established, and the laboratory evidence is overwhelming, while the human evidence amounts to a handful of trials. That gap is itself the finding: the field has not, to date, treated fermented foods as bile acid–modulating therapies worth measuring as such.

### 3.3 Dietary Fat Composition

The best‐established disease association in bile acid biology, linking the Western diet to secondary bile acids and colorectal cancer, runs through dietary fat [16, 37]. Diets that are rich in saturated fat and red meat protein and depleted in fiber and micronutrients increase bile acid synthesis in the liver. This in turn elevates bile acid concentrations in the gut and shifts the secondary bile acid pool toward higher concentrations of DCA. DCA acts as a tumor promoter through multiple mechanisms. It activates *β*‐catenin and Wnt signaling, a pathway that drives cell proliferation at low micromolar concentrations [38]. At higher concentrations, it has been associated with epithelial–mesenchymal transition, a process in which epithelial cells lose aspects of their normal organization and acquire features associated with invasion and motility. With prolonged exposure, DCA also produces direct mutagenic effects on the colonic epithelium [39].

The mechanistic data are more complex than a model based only on increased luminal bile acid exposure. Long‐term studies in mice fed a Western diet have shown that diet‐induced colorectal cancer does not result from bile acid overproduction alone. It results from the combination of elevated bile acid exposure in the gut and impaired bile acid transport into the colon's lining cells [37]. Western diet feeding increases colon tumor numbers and bile acid concentrations in the gut as expected, but it simultaneously reduces bile acid concentrations inside the colon's lining cells and downregulates the bile acid transporters FABP6 and OSTβ, along with ASBT. The corresponding reduction in FXR signaling inside cells removes a normal brake on cell proliferation [37]. The net result is high bile acid concentrations in the gut lumen paired with low FXR‐mediated restraint inside the cells lining the colon, a combination far more dangerous than either feature in isolation. The therapeutic implication is that dietary interventions combining multiple foods or food components into a single therapy are mechanistically well‐matched to the actual pathology. Fiber can reduce bile acid exposure in the gut. Fermented foods and polyphenol‐rich foods could be tested for their ability to shift secondary bile acid balance. Shifts in dietary fat composition can reduce the synthesis drive in the liver. This pattern points to a basic limitation of drug development for bile acid–driven disease: a drug that activates a single receptor can only address one point in this system, whereas a coordinated dietary strategy can address several at once.

Large studies in people support the same conclusion. In the European Prospective Investigation into Cancer and Nutrition cohort, researchers measured bile acid levels in blood samples that had been collected from healthy participants years before any cancer diagnosis. Participants with higher plasma bile acid levels at baseline went on to develop colon cancer at higher rates than those with lower levels [40]. This is the kind of evidence animal studies cannot produce on their own, because it traces what happens in human populations over time rather than what can be induced in mice. Despite this evidence, bile acid‐guided dietary or fermented food interventions have not become standard prevention strategies for patients at elevated colorectal cancer risk.

### 3.4 Polyphenols and Protein Source

Two additional dietary inputs deserve brief mention because they illustrate the broader pattern. Polyphenols, a class of plant‐derived compounds found in tea and berries, along with many other foods, modulate bile acid metabolism through two parallel routes. They directly bind bile acids in the small intestinal lumen, and they reshape the microbial community in ways that alter BSH expression and downstream secondary bile acid formation [41]. The bidirectional nature of polyphenol effects is itself instructive: the same polyphenol can inhibit or stimulate BSH activity depending on its specific structure and the microbial context in which it acts. This pattern reflects what successful bile acid–targeted therapeutics will look like in the future, as they will be interventions that shift the composition of the bile acid pool rather than drugs that activate a single receptor.

Dietary protein source represents the newest and least developed portion of this story. Prior work suggests that animal‐based diets can increase fecal bile acids and favor bile‐tolerant taxa, including Alistipes, Bilophila, and Bacteroides, while reducing Firmicutes associated with plant‐polysaccharide fermentation [42]. The newer question is how dietary protein shapes MCBA production: because BSH operating in re‐conjugation mode requires both a bile acid and an amino acid substrate, the colonic amino acid pool could plausibly determine which MCBAs the community produces. To my knowledge, this hypothesis has not been tested directly in humans. It illustrates the broader point of this section: the bile acid pool is the variable through which several large but disconnected dietary literatures could be connected.

## 4 Where Bile Acid Pathways Already Drive Disease

Four gastrointestinal conditions illustrate the gap between what is known about bile acids and what gets done with that knowledge in clinical practice. These are also the conditions where dietary intervention has the strongest case as a targeted therapy.

### 4.1 Irritable Bowel Syndrome With Bile Acid Diarrhea

Roughly a quarter to a third of patients diagnosed with IBS‐D have undiagnosed BAD [13, 14]. In this condition, excess bile acids reach the colon and pull water into the gut lumen, producing the diarrhea, urgency, and quality‐of‐life impact that the patient initially attributes to IBS. BAD is treatable, and the diagnostic tests already exist. Fasting measurement of FGF19 and selenium‐75 homocholic acid taurine (75SeHCAT) retention testing are both clinically validated [13, 43]. Yet the diagnosis is often missed in practice [13, 43].

The first‐line treatment is to bind bile acids in the gut using cholestyramine or colestipol, along with colesevelam, all drugs introduced in the 1960s and 1970s. Current guidelines center on diagnosis and bile acid sequestration; they do not include an FGF19‐mimetic strategy, a BSH‐targeted probiotic therapy, or a specified dietary intervention as standard treatment [13, 15]. This is despite a strong mechanistic case for combining fiber‐based bile acid binding with microbiome modulation through fermented foods.

The clinical mechanism is well‐known, and the patient population is large. The standard of care is sufficiently inadequate that this population should be the first testing ground for specified dietary therapeutics evaluated with bile acid outcomes.

### 4.2 Post‐Cholecystectomy Syndromes

Cholecystectomy, or surgical removal of the gallbladder, is among the most common abdominal surgeries performed in the developed world, with certain adult populations approaching 10% [44]. Under normal physiology, the gallbladder stores bile between meals and releases it in concentrated pulses when food enters the small intestine. After removal of the gallbladder, this pulsed delivery is converted into a continuous small trickle of bile into the duodenum. The result is a long‐term readjustment of the bile acid pool, including chronically elevated bile acid exposure in the colon and a microbial community that shifts toward bacteria that tolerate bile, along with elevated concentrations of secondary bile acids [44, 45].

Testing of post‐cholecystectomy patients investigated for diarrhea has identified BAD in approximately 60% [15], making this population one of the clearest identifiable groups in which BAD should be considered. Post‐cholecystectomy patients have also been investigated for elevated colorectal cancer risk, given the chronically elevated colonic secondary bile acid exposure mentioned above. Epidemiologic evidence remains scattered, with earlier meta‐analyses suggesting a minor risk increase and recent updated analyses finding no significant association [46].

The colorectal cancer data should change how cholecystectomy is interpreted in bile acid research. Cholecystectomy alters the timing and pattern of bile delivery and can increase colonic bile acid exposure, but it does not appear, by itself, to be a major colorectal cancer risk state. That distinction matters. Secondary bile acid exposure depends not only on bile delivery, but also on diet, transit time, microbial 7α‐dehydroxylation capacity, bile acid reabsorption, and regional differences across the colon. Cholecystectomy is therefore an imperfect proxy for the exposure that matters most: the measured composition and concentration of the colonic bile acid pool. The absence of a convincing overall colorectal cancer association strengthens the case for measuring bile acids directly rather than relying on surgical history alone.

The therapeutic response to post‐cholecystectomy bile acid dysregulation has also been limited. Patients with diarrhea after cholecystectomy are often treated empirically with the same bile acid binders developed decades ago, sometimes without explicit diagnostic confirmation. No targeted dietary intervention exists in clinical guidelines, and no microbiome‐based therapeutic addresses the underlying shift in bile acid pool composition. This population is therefore a strong case for routine bile acid evaluation and for trials of specified dietary therapy as a primary intervention.

### 4.3 Cholestatic and Metabolic Liver Disease

In cholestatic and metabolic liver disease, therapeutic development has focused most directly on hepatic bile acid signaling. OCA, the leading FXR‐activating drug, was approved in 2016 for PBC [47]. It was later rejected by the FDA for non‐alcoholic steatohepatitis in 2020 despite meeting histologic improvement endpoints in REGENERATE [9], rejected again on resubmission in 2023 [10], received an FDA safety communication for serious liver injury in 2024 [11], and was withdrawn from the United States market in 2025 [12].

For receptor‐directed bile acid pharmacology, the central challenge is separating therapeutic receptor activation from concentration‐dependent toxicity in disease populations where bile acid handling is already impaired.

The microbiome side of the story offers a different path. Approximately 30%–40% of patients with PBC respond inadequately to ursodeoxycholic acid (UDCA), the standard first therapy [19, 47]. The gut bacteria of these non‐responders differ from responders in consistent ways, including lower abundance of BSH genes and reduced microbial diversity. Probiotic interventions in animal models of cholestatic liver disease have shifted bile acid composition and reduced liver injury [19]. Mediterranean and gluten‐free dietary interventions in small human trials of cholestatic disease have reduced inflammatory markers and symptom scores [19]. FMT in pilot studies of primary sclerosing cholangitis has produced meaningful reductions in alkaline phosphatase, a marker of bile duct injury, in a subset of patients [48].

None of these interventions has replaced UDCA. Where direct receptor pharmacology has failed, microbial modulation of the bile acid pool is producing signal. Drugs that activate the PPARs are emerging as effective second‐line therapies [17, 18], but they work in parallel to the bile acid pathway rather than through it. One plausible path for future bile acid–targeted therapy in cholestatic and metabolic liver disease is microbial modulation of the bile acid pool, and the recent withdrawal of OCA should be read as a clarification rather than a setback.

### 4.4 Colorectal Cancer

The bile acid mechanism in colorectal cancer is arguably the longest‐standing example of a mechanism without translation into a prevention strategy [16, 38]. The epidemiologic evidence has been established for years [40], and the dietary interventions that would address the underlying pathology have been described for decades. Yet they are not standard recommendations for elevated‐risk patients. Aspirin appears in some guidelines; fiber supplementation is rarely mentioned; and fermented food consumption is almost entirely absent from formal recommendations. The interventional studies needed to translate the bile acid mechanism into prevention strategies have not been conducted at the scale that translation would require. This is precisely the gap that routine bile acid outcomes in nutrition research would help close.

## 5 Dietary Therapy With the Precision of a Drug

Four therapeutic approaches are available for intervening in the bile acid system. They are at different stages of development and require different kinds of infrastructure to advance. This Perspective argues that the most immediately implementable, and the one most relevant to nutrition science, is dietary therapy designed and evaluated with measurable bile acid outcomes. The three other approaches deserve brief mention as the broader landscape in which dietary therapy fits.

The first is engineered probiotics and defined microbial mixtures designed to perform specific bile acid chemistry. Work on BSH activity and substrate specificity [27, 32] suggests that strains can be selected or engineered to produce specific bile acid pool compositions. The FDA‐approved defined microbial products for recurrent *C. difficile* infection, SER‐109 and RBX2660, demonstrate that this approach can reach the clinic [21, 49].

The second is direct pharmacology based on MCBAs themselves. This approach is the earliest in development, but it has the highest ceiling because the field has identified a catalog of perhaps several hundred MCBAs whose biological activities are largely uncharacterized and awaiting screening [7, 8, 26].

The third is FMT. This approach is the most clinically tested but the most narrowly applicable. The recurrent *C. difficile* template is established [21], and emerging evidence supports its use in primary sclerosing cholangitis [48], but its applicability is limited to conditions where the bile acid mechanism is explicit.

Dietary therapeutics differ from these three approaches in a fundamental way: the interventions already exist. Soluble fiber and fermented foods, along with polyphenol‐rich foods, are not awaiting development, just recognition as targeted bile acid therapy.

The framing matters more than it might seem. Dietary recommendations in clinical gastroenterology are currently delivered as lifestyle advice. Patients are told to eat more fiber, consume fermented foods, and follow a Mediterranean diet. These recommendations are mechanistically sound but clinically weak because they lack the precision that a pharmacological intervention would need. A patient with BAD is not told to consume 30 g of psyllium daily, timed to peak bile acid secretion to maximize binding in the small intestine and minimize colon spillover. A patient with PBC is not given a polyphenol formulation designed to engage the FXR–FGF19 signaling loop through changes in pool composition. The interventions exist; they are simply not deployed with therapeutic specificity.

This is a problem of how the field has categorized dietary intervention, not a clinical limitation. The framing of dietary intervention as lifestyle advice rather than therapy is a consequence of an earlier era of nutrition science, when mechanism was poorly characterized, and clinical effects could only be measured in broad averages across large populations. Modern bile acid biology has changed this, as the mechanism is now characterized at the level of individual enzymes and individual metabolites. The measurement infrastructure exists, the patient populations who would benefit are identifiable, but what is required is the clinical and regulatory framework to treat dietary interventions as the targeted therapies they have become.

Two concrete trials would test this framing directly and would be unmistakably interpretable. The first is a randomized trial of soluble fiber timed to bile acid secretion in post‐cholecystectomy patients with elevated BAD symptoms. The primary outcome would be bile acid excretion in stool, with symptom scores and quality of life as secondary outcomes. The second is a trial of fermented food intervention in patients with PBC whose response to UDCA is inadequate. The primary outcome would be the composition of the bile acid pool, with alkaline phosphatase as a secondary outcome. Neither trial is exotic, but neither has, to my knowledge, been conducted with the design specificity that the mechanism supports. The infrastructure already exists, but what is missing is the recognition that these are clinical pharmacology trials of dietary therapeutics, not lifestyle intervention studies.

Dietary therapeutics will not be developed by the pharmaceutical industry. If they are developed at all, they will be developed by academic medical centers in collaboration between dietitians and physicians. The case for treating fermented foods and specific fibers, along with polyphenol‐rich foods, as targeted bile acid therapy is the central claim of this Perspective. It is a claim that nutrition science is uniquely positioned to advance.

## 6 A Research Agenda for Bile Acid Integration

The biology supports treating bile acids as the central variable for research that connects diet, the gut microbiome, and disease. The clinical need is relevant, and the technical infrastructure largely exists. Four specific recommendations follow:

First, bile acid outcomes should be incorporated into the standard reporting panel for diet and microbiome intervention studies. Dietary research has split into separate literatures organized around fiber, fermented foods, polyphenols, and macronutrient composition. Each community has built its own evidence in isolation. The bile acid pool is where these separate literatures should converge. Routine bile acid reporting would, over a decade, produce the integrated dataset the field currently lacks. The technical barriers to this change are increasingly manageable because targeted LC–MS/MS bile acid profiling is now widely used in metabolomics studies. LC–MS panels for major bile acid species are increasingly available, especially in studies already using metabolomic infrastructure [7, 8, 50]. The statistical tools for comparing bile acid measurements with microbial community data are well‐established and widely used [7, 8, 31]. Journals that publish microbiome and dietary research should treat bile acid outcomes the way they currently treat short‐chain fatty acid outcomes: as something a serious study is expected to measure.

Second, clinical gastroenterology practice should incorporate bile acid evaluation into the routine workup of conditions in which the bile acid mechanism is established. Patients with IBS‐D should be screened for BAD using fasting FGF19 or 75SeHCAT testing [13, 43]. This screening matters because a quarter to a third of these patients have a treatable bile acid disorder that goes undiagnosed under current practice. Patients with symptoms after gallbladder removal should receive a directed bile acid evaluation rather than being treated observationally with antidiarrheal medications. Patients with PBC whose response to UDCA is inadequate should have the composition of their bile acid pool characterized before a second‐line drug is selected. The diagnostic tests for these workups largely exist; what the clinical workflow does not yet include is the habit of using them.

Third, the regulatory and developmental framework for dietary therapeutics needs to improve. Dietary interventions designed with the precision that pharmacological therapy requires fall awkwardly between the existing categories. They are not medical foods in the traditional sense, and they are not conventional supplements. Trials of soluble fiber timed to bile acid secretion in post‐cholecystectomy patients, or of defined fermented food protocols in PBC, will require regulatory innovation comparable to what the *C. difficile* defined microbial products required to reach approval. This problem is solvable, but solving it requires nutrition researchers and regulators to decide together what an approval pathway for dietary therapies should look like.

Fourth, the measurement and monitoring infrastructure for bile acids should be developed at a scale appropriate to the disease populations involved. The bile acid pool is not a static variable; it fluctuates with diet, time of day, and the state of the microbiome in the gut. It shifts over weeks to months in response to dietary or pharmacological intervention, and it is most informative when measured repeatedly over time rather than at a single moment. At‐home stool collection and shipment infrastructure, of the kind that direct‐to‐consumer microbiome companies have begun to deploy, could make repeated bile acid monitoring plausible at large scale. The implications for both research and clinical care have not yet been fully appreciated by the field.

The history of how SCFAs became the standard set of molecules used to interpret dietary fiber effects offers a useful precedent. Twenty years ago, butyrate, propionate, and acetate were research compounds measured in a small number of specialist labs. The integration of SCFA measurement into standard microbiome workflows occurred gradually, driven by accumulating evidence that these molecules mediated effects too important to leave unmeasured. Bile acids are analytically more complex than SCFAs, requiring LC–MS rather than the simpler gas chromatography sufficient for SCFAs. This represents a real but manageable barrier. The same kind of integration is now available to bile acid biology, although the analytical complexity is greater. The field's task is to recognize the similarity and act on it.

Bile acids are a crucial variable for advancing diet–microbiome research into clinically testable nutrition science. Treating them as measurable therapeutic targets may help move dietary intervention from general advice toward the next generation of clinical nutrition.

## Author Contributions

Jason Dichter conceived and designed the work, conducted the literature synthesis, and drafted and revised the manuscript. As the sole author, Jason Dichter takes full responsibility for the integrity and content of the work.

## Funding

The author has nothing to report.

## Conflicts of Interest

The author declares no conflicts of interest.

## Data Availability

Data sharing is not applicable to this article as no datasets were generated or analyzed during the current study.
